# Neuroprotective Effect of Aurantio-Obtusin, a Putative Vasopressin V_1A_ Receptor Antagonist, on Transient Forebrain Ischemia Mice Model

**DOI:** 10.3390/ijms22073335

**Published:** 2021-03-24

**Authors:** Pradeep Paudel, Dong Hyun Kim, Jieun Jeon, Se Eun Park, Su Hui Seong, Hyun Ah Jung, Jae Sue Choi

**Affiliations:** 1 Department of Food and Life Science, Pukyong National University, Busan 48513, Korea; phr.paudel@gmail.com (P.P.); gogo1685@naver.com (S.E.P.); seongsuhui@naver.com (S.H.S.); 2 National Center for Natural Products Research, Research Institute of Pharmaceutical Sciences, The University of Mississippi, Oxford, MS 38677, USA; 3 Department of Health Sciences, The Graduate School of Dong-A University, Busan 49315, Korea; mose79@dau.ac.kr (D.H.K.); ji6785@naver.com (J.J.); 4 Department of Biomedical Science, Asan Medical Institute of Convergence Science and Technology, Seoul 05505, Korea; 5 Department of Food Science and Human Nutrition, Jeonbuk National University, Jeonju 54896, Korea

**Keywords:** cassia, aurantio-obtusin, vasopressin receptor, antagonist, molecular docking

## Abstract

Traditional Chinese medicines (TCMs) have been a rich source of novel drug discovery, and Cassia seed is one of the common TCMs with numerous biological effects. Based on the existing reports on neuroprotection by Cassia seed extract, the present study aims to search possible pharmacological targets behind the neuroprotective effects of the Cassia seeds by evaluating the functional effect of specific Cassia compounds on various G-protein-coupled receptors. Among the four test compounds (cassiaside, rubrofusarin gentiobioside, aurantio-obtusin, and 2-hydroxyemodin 1-methylether), only aurantio-obtusin demonstrated a specific V_1A_R antagonist effect (71.80 ± 6.0% inhibition at 100 µM) and yielded an IC_50_ value of 67.70 ± 2.41 μM. A molecular docking study predicted an additional interaction of the hydroxyl group at C6 and a methoxy group at C7 of aurantio-obtusin with the Ser341 residue as functional for the observed antagonist effect. In the transient brain ischemia/reperfusion injury C57BL/6 mice model, aurantio-obtusin attenuated the latency time that was reduced in the bilateral common carotid artery occlusion (BCCAO) groups. Likewise, compared to neuronal damage in the BCCAO groups, treatment with aurantio-obtusin (10 mg/kg, p.o.) significantly reduced the severity of damage in medial cornu ammonis 1 (mCA1), dorsal CA1, and cortex regions. Overall, the findings of this study highlight V_1A_R as a possible target of aurantio-obtusin for neuroprotection.

## 1. Introduction

Vasopressin (AVP) and oxytocin have been implicated in the etiology of psychiatric disorders, such as schizophrenia [1], autism [2,3,4], and depression [5]. AVP acts centrally within the central nervous system (CNS) where it modulates a range of behaviors from learning and memory and responses to stressors to social behaviors [6]. The vasopressin 1A receptor (V_1A_R) has been contemplated to play the dominant role in regulating behavior and until recently, among vasopressin subtypes (V_1A_, V_1B_ and V_2_); the V_1A_R is thought to be the only subtype expressed widely in the brain [6,7,8]. A recent study showed a marked reduction in anxiety-like behavior and a profound impairment in social recognition in V_1A_R knock-out (V_1A_RKO) mice [9]. Similarly, a study by Ferris et al. [10] suggested that V_1A_ receptor antagonists may be used to treat interpersonal violence co-occurring with an illness such as attention-deficit/hyperactivity disorder, autism, bipolar disorder, and substance abuse. Therefore, it is hypothesized that antagonistic action on the V_1A_ receptor might attribute to the treatment approach in anxiety-like behavior and recently, discovery of a potent, selective, and brain penetrant V_1A_ receptor antagonist is emerging [4,11]. Additionally, AVP had been reported to mediate brain edema formation and cerebral ischemia by regulating water permeability in astrocytes [12]. Similarly, the peripheral role of V_1A_R in the inflammatory process of inflammatory bowel disease (IBD) mediated by prostaglandin release has been reported recently [13]. In the published report, V_1A_R promoted COX-2-dependent prostaglandin release from a mucosal mast in 2,4,6-trinitrobenzene sulfonic acid (TNBS)-induced colitis in mice, which was attenuated by conivaptan (a V_1A_R antagonist).

Besides, AVP has numerous peripheral roles. Linas and colleagues [14] had previously reported an increased AVP level along with impaired renal water excretion and abnormal renal hemodynamics in a mouse model of CCl_4_-induced liver cirrhosis. Similarly, a recent study on the ischemia-reperfusion injury mouse model [15] identified upregulated V_1_R expression in hepatocytes and highlighted the importance of the hepatocyte V_1_R/Wnt/β-catenin/FoxO3a/Akt pathway in hepatoprotection. Additionally, V_1A_R in vascular smooth muscles is responsible for vasoconstriction, myocardial contractility, platelet aggregation, and uterine contraction [16,17] and regulates blood pressure in vascular walls [18,19]. It has been reported previously that AVP is released in response to peripheral inflammation [20,21] which is deleterious to various immune-mediated diseases. A recent report on the Phase II clinical trial of a selective V_1A_R antagonist balovaptan suggested it as a potential treatment for the socialization and communication deficits in autism spectrum disorder [4,22]. Likewise, investigations on the functional role of V_1A_R in cardiovascular homeostasis using gene targeting demonstrated lower basal blood pressure in mutant mice lacking the V_1A_R gene (V_1A_^−/−^) compared to the wild-type mice (V_1A_^+/+^) [18,23].

**Figure 1:**
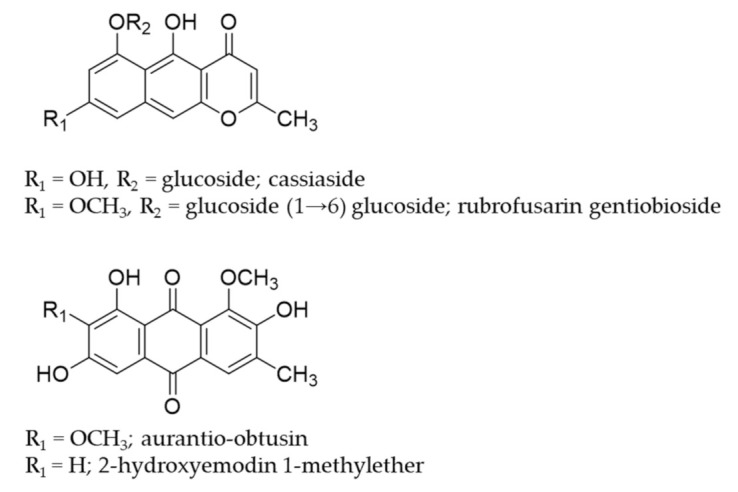
Structures of compounds isolated from Cassia seeds.

Cassia seed has a long history of use in brewing tea in South Korea. Additionally, in traditional Chinese medicine, Cassia seeds have been used as vision improving, aperient, diuretic, antiasthenic, and an effective agent in lowering cholesterol and reducing blood pressure along with anthraquinones and naphthopyrones derivatives as predominant constituents, particularly the glycosides (cassiaside and rubrofusarin gentiobioside) [24]. Likewise, a comparison of HPLC chromatograms of various anthraquinones from Cassia seeds, revealed aurantio-obtusin as a predominant aglycon [25]. The major biological activities of aurantio-obtusin reported so far include antihypertensive [26], hepatoprotective [27], antimutagenic [28], osteogenic [29], and estrogenic activities [30]. Cassia seeds extract and its constituents have been reported for the management of various diseases including Alzheimer’s disease [31,32,33,34], Parkinson’s disease [35], diabetes and diabetic complications [36,37], hepatotoxicity [38,39], inflammation [34], oxidative stress [40,41], and many more [42]. Similarly, seeds extract has been reported as a therapy for neurodegenerative disorders in recent years [43]. However, there exist only a few reports on the effects of specific compounds present in Cassia seeds. More recently, we have discovered anthraquinones as promising human monoamine oxidase-A (hMAO-A) inhibitors [44], and emodin and alaternin (=7-hydroxyemodin) as potent vasopressin V_1A_R antagonists and dopamine D_3_R agonists [45]. In this study, we evaluated the functional effect of cassiaside, rubrofusarin gentiobioside, and aurantio-obtusin (Figure 1) on vasopressin V_1A_ receptor, 5-HT_1A_ receptor, neurokinin receptor, and dopamine D3 receptor because these receptors were predicted as top protein targets of cassia-derived compounds in neurodegenerative diseases [45]. Additionally, the effect of aurantio-obtusin has been compared with 2-hydroxyemodin 1-methylether for the elucidation of structure–activity relationship and the probable mechanism of ligand–receptor interaction was assessed via molecular docking simulation. Besides, we report herein the neuroprotective effect of aurantio-obtusin in a transient forebrain ischemia mice model.

## 2. Results

### 2.1. Functional Effect of Test Compounds via cAMP Modulation

The functional role (agonist and antagonist effect) of test compounds (Figure 1) on dopamine receptors was assessed by measuring their effect on cAMP modulation in transfected Chinese hamster ovary (CHO) cells using homogeneous time-resolved fluorescence (HTRF) detection. As shown in Table 1, only aurantio-obtusin showed mild agonist effect by stimulating the dopamine effect by 33.00 ± 1.84% (*p* < 0.05).

However, cassiaside and rubrofusarin gentiobioside demonstrated a negligible agonist effect on hD_3_R. The compounds stimulated the effect of dopamine (300 nM) by 14.75 ± 1.20 and 7.05 ± 1.63%, respectively. The reference control agonist, dopamine exhibited an EC_50_ value of 2.7 nM. However, none of the test compounds showed an antagonist effect. The percentage inhibition of agonist response of dopamine (10 nM) by the test compounds was negative for hD_3_R. Reference antagonist (+)-butaclamol exhibited an IC_50_ value of 25 nM.

### 2.2. Functional Effect of Test Compounds via Intracellular Ca^2+^ Ion Modulation

**Figure 2:**
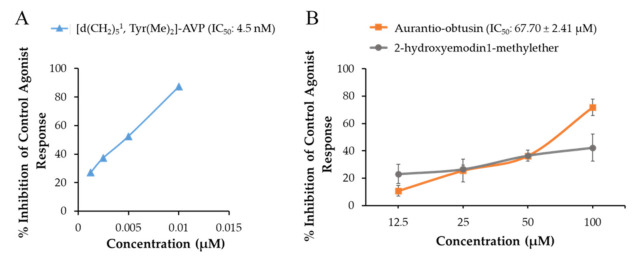
Concentration-dependent vasopressin V_1A_R antagonist effect of (**A**) reference antagonist (d(CH_2_)5^1^,Tyr(Me)_2_]-vasopressin (AVP)) and (**B**) active compounds from Cassia seeds (aurantio-obtusin and 2-hydroxyemodin 1-methylether). Reference drugs and test samples were tested at the indicated concentration for antagonist effect by determining the percentage inhibition of control response to 10 nM AVP. (**B**) Represents a comparative inhibition pattern of aurantio-obtusin with its substructure, 2-hydroxyemodin 1-methylether. Values are expressed as mean ± SD of triplicate experiment.

The functional effect (agonist and antagonist effect) of test compounds on the h5-HT_1A_R, hNK_1_R, and hV_1A_R was evaluated by measuring the intracellular Ca^2+^ concentration. As shown in Table 1, the agonist response of test compounds on the tested receptors was negligible at 100 µM (% stimulation of control agonist effect by the test compounds was negligible). Likewise, the antagonist effect of cassiaside and rubrofusarin gentiobioside was also negligible. Only aurantio-obtusin demonstrated antagonist effect on hNK_1_R (47.60 ± 3.11% at 100 µM; *p* < 0.01) and V_1A_R (71.80 ± 6.08% at 100 µM; *p* < 0.001). Since the V_1A_R antagonist effect of aurantio-obtusin was greater than 70% at 100 µM, the concentration-dependent antagonist effect was evaluated at various concentrations up to 100 µM and the 50% inhibition concentration (IC_50_ value) was determined. Additionally, we tested the V_1A_R antagonist effect of 2-hydroxyemodin 1-methylether which is a substructure of aurantio-obtusin. As shown in Figure 2, aurantio-obtusin showed a concentration-dependent antagonist effect with an IC_50_ value of 67.70 ± 2.41 µM. Likewise, 2-hydroxyemodin 1-methylether also showed a concentration-dependent antagonist effect with 23.1%, 26.5%, 36.6%, and 42.3% inhibition at 12.5, 25, 50, and 100 µM, respectively. Reference antagonist [d(CH_2_)5^1^,Tyr(Me)_2_]-AVP demonstrated an IC_50_ value of 4.5 nM.

### 2.3. Molecular Docking Simulation

**Figure 3:**
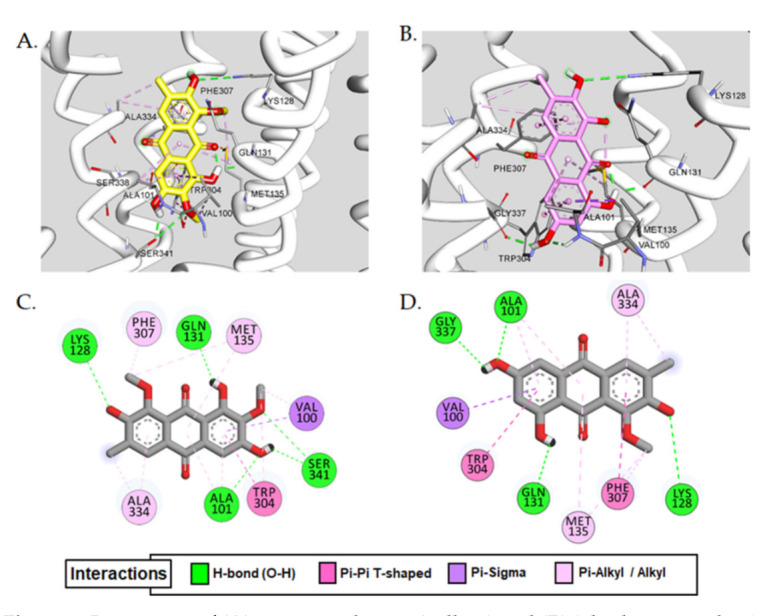
Interaction of (**A**) aurantio-obtusin (yellow) and (**B**) 2-hydroxyemodin 1-methylether (pink) in the active site of vasopressin V_1A_ receptor (V_1A_R). (**C**,**D**) represent 2D-binding diagrams of aurantio-obtusin and 2-hydroxyemodin 1-methylether in V_1A_R, respectively. Dotted-colored lines represent interaction types—green: H-bond interaction; maroon: π-π T-shaped; purple: π-sigma; pink: π-alkyl/alkyl interaction.

Aurantio-obtusin–V_1A_R interaction was analyzed via molecular docking simulation using AutoDock 4.2 and the mechanism was compared with its substructure, 2-hydroxyemodin 1-methylether along with the reference ligands. Overall docking results (binding score and interacting residues) are depicted in Figure 3 and tabulated in Table 2.

As can be seen in Table 2, both aurantio-obtusin and 2-hydroxyemodin 1-methylether were predicted to bind at the active site of V_1A_R with a lower binding score of approximately −7.5 kcal/mol compared to the reference agonist AVP (−5.8 kcal/mol) and antagonist (−6.07 kcal/mol). Common H-bond interactions observed for both the test compounds were at Ala101 and Gln131 with methoxy group at C6, and at Lys128 with the hydroxyl group at C2 (Figure 3C,D). Additionally, Gln131 and Lys128 were involved in H-bond interaction with the reference antagonist d(CH_2_)5^1^,Tyr(Me)_2_]-AVP. However, in addition to the H-bond interacting residues, the hydroxyl group at C6 and a methoxy group at C7 of aurantio-obtusin showed multiple interactions with the Ser341 (Figure 3A,C). Other common hydrophobic residues involved in test ligand-V_1A_R interaction were Val100(π-sigma), Trp304(π-π T-shaped, π-alkyl), Ala334(alkyl, π-alkyl), Met135(alkyl, π-alkyl), Val100(alkyl), Phe307(π-alkyl), and Ala101(π-alkyl) (Table 2 and Figure 3C).

### 2.4. Drug-Likeness and ADME Prediction

Drug-likeness was predicted for aurantio-obtusin and the prediction results are tabulated in Table 3. As shown in the table, aurantio-obtusin exhibited good drug-like properties. It was predicted mid-structure according to the MDDR-like rule [46] and considered as a suitable drug candidate molecule based on the Lipinski’s rule [47].

According to absorption, distribution, metabolism, and excretion (ADME) prediction, moderate plasma protein binding (86.98%), good human intestinal absorption (84.66%), and good lipophilicity (2.53) were predicted. As reviewed earlier [48], lipophilicity (Log Po/w) value in the range of 1.5–2.5 indicates the suitability for CNS delivery. Likewise, the blood–brain barrier (BBB) penetration value ([brain]/[blood]) was 0.48% indicating moderate absorption by the CNS. All these predicted results could be utilized for optimizing drug-like properties. Additionally, permeability across Madin–Darby Canine Kidney (MDCK) and human epithelial colorectal adenocarcinoma (Caco2) cells was predicted to be 113.20 and 19.19 nm/s, respectively.

### 2.5. Neuroprotective Effect of Aurantio-Obtusin

**Figure 4:**
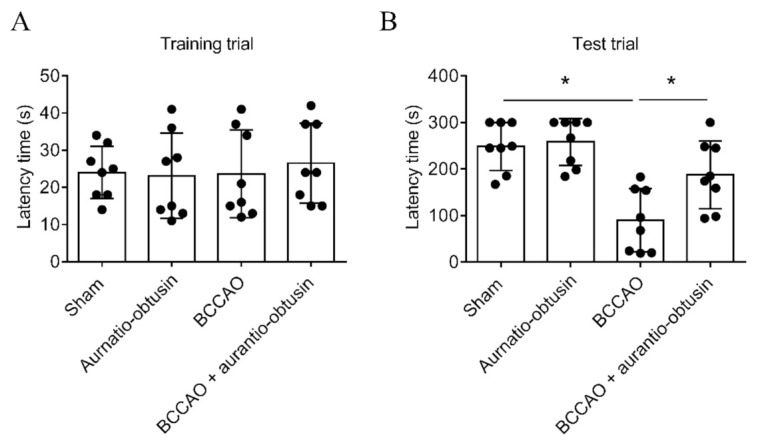
Effect of aurantio-obtusin on transient forebrain ischemia-induced memory impairment in a passive avoidance test. (**A**) Latency time in training trial of passive avoidance test. (**B**) Latency time in test trial of passive avoidance test. Data represented as mean ± SD with raw data. * *p* < 0.05.

**Figure 5:**
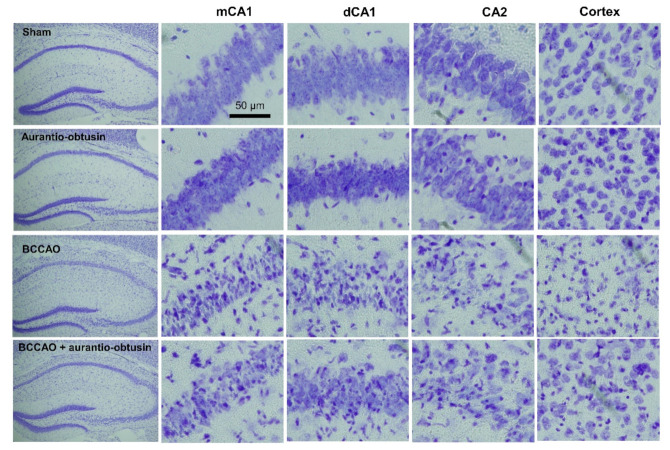
Photomicroscopes of Nissl staining showing degree of transient forebrain ischemia-induced neuronal damage in different brain regions.

**Figure 6:**
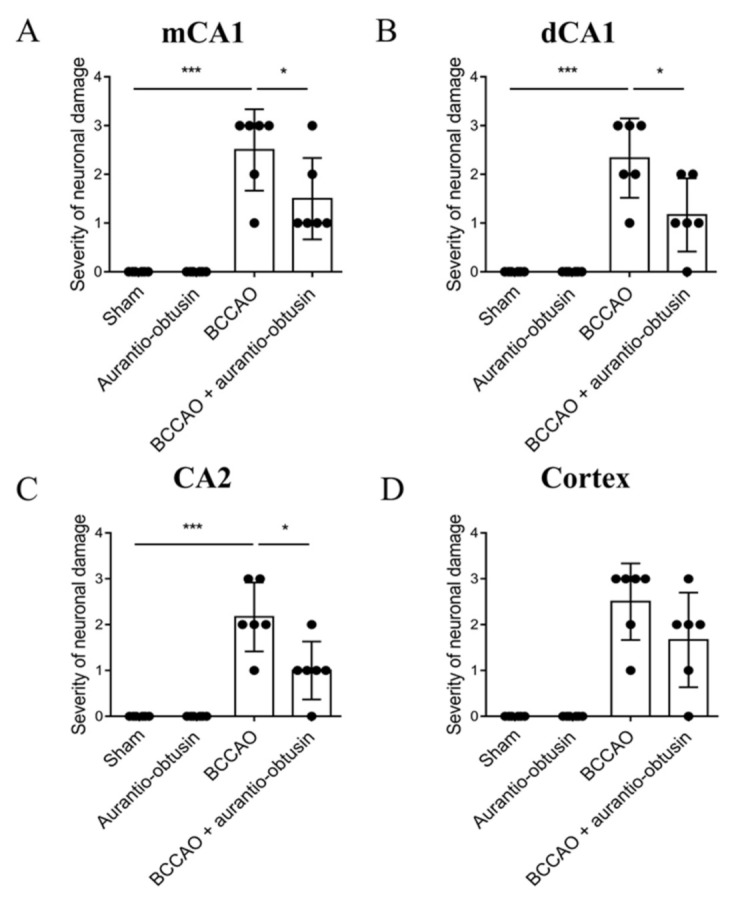
Effect of aurantio-obtusin on transient forebrain ischemia-induced neuronal damage. (**A**−**D**). Severity of neuronal damage in mCA1 (**A**), dCA1 (**B**), CA2 (**C**), and cortex (**D**) regions. The cells were counted in six sections by every eight sections interval (total 48 sections) per animal by a person blind to the treatment group, and the average cell count per section was computed. The degree of damage by the Nissl staining after ischemia was semiquantitatively scored from 0 to 3. Neurons showing whole neuronal body shape were determined as healthy neurons. The percentage of healthy neurons compared to sham group was used as quantification criteria. (0, same to shame group; 1, >70% of sham group; 2, 40–70% of sham group; 3, 0–40% of sham group). Data represented as mean ± SD with raw data. * *p* < 0.05, *** *p* < 0.001.

As V_1A_R antagonist has neuroprotective effect against ischemic brain damage, we tested aurantio-obtusin on a transient forebrain ischemia mice model. In a pilot study, we tested 1, 5 and 10 mg/kg of aurantio-obtusin in bilateral common carotid artery occlusion (BCCAO) model, and we found that 10 mg/kg of aurantio-obtusin is effective. Therefore, we selected 10 mg/kg of aurantio-obtusin in the main in vivo experiment. In the training trial of the passive avoidance test, there were no significant differences in latency time between groups (*F*_3,28_ = 0.1654, *p* > 0.05, *n* = 8/group, Figure 4A). BCCAO groups showed significant reduction in latency time in test trial of passive avoidance test (*p* < 0.05). Aurantio-obtusin significantly attenuated the reduction of latency time by BCCAO (*F*_3,28_ = 12.67, *p* < 0.05, *n* = 8/group, Figure 4B). To observe neuronal damage, Nissl staining was conducted (Figure 5). BCCAO group showed significant increase in severity of neuronal damage compared to sham group in medial cornu ammonis 1 (mCA1), dorsal CA1 (dCA1), CA2, and cortex regions. Aurnatio-obtusin significantly reduced the severity in mCA1, dCA1, and cortex regions, but not in CA2 region (mCA1, *F*_3,20_ = 25.71, *p* < 0.05, Figure 6A; dCA1, *F*_3,20_ = 24.28, *p* < 0.05, Figure 6B; CA2, *F*_3,20_ = 21.23, *p* < 0.05, Figure 6C; cortex, *F*_3,20_ = 26.38, *p* < 0.05, Figure 6D, *n* = 6/group).

## 3. Discussion

In this study, we tested functional effect of cassiaside, rubrofusarin gentiobioside, and aurantio-obtusin on V_1A_R, D_3_R, NK_1_R, and 5-HT_1A_R which were the top protein targets for Cassia compounds in neurodegenerative diseases predicted via proteocheminformatics modeling [45]. Among the tested compounds, only an anthraquinone aurantio-obtusin showed a good V_1A_R antagonist effect. Naphthopyrone glycosides cassiaside and rubrofusarin gentiobioside remained inactive on these receptors. Quinone derivatives were previously predicted as reactive and pan assay interference compounds (PAINS) through high throughput screening that could show false biological activities [49,50]. If so, then all quinones should show activity. Therefore, we compared the V_1A_R antagonist effect of aurantio-obtusin with its substructure 2-hydroxyemodin 1-methylether (Figure 2). Both compounds are anthraquinones but only aurantio-obtusin showed good antagonist effect while 2-hydroxyemodin 1-methylether showed mild V_1A_R antagonist effect (42.3% inhibition at 100 μM concentration). Likewise, in our recent report on G-protein-coupled receptors modulation by anthraquinones from Cassia seed [45], only emodin and 7-hydroxyemodin showed activity on dopamine and vasopressin receptors. Other anthraquinones aloe-emodin and questin remained inactive at the tested concentrations. Therefore, the SAR would be evidence that all quinones are not PAINS [49].

Comparison of the V_1A_R antagonist effect of anthraquinones emodin and alaternin (=7-hydroxy emodin) with other anthraquinnones from our recent study [45] revealed that the hydroxyl group at C1, C3 and C8 and a methyl group at C6 of anthraquinone structure are essential for the hV_1A_R antagonist effect. In this study, we tested aurantio-obtusin (1,3,7-trihydroxy-2,8-dimethoxy-6-methylanthracene-9,10-dione) which differs slightly from emodin and alaternin for its functional effect on V_1A_R and compared with its sustructure 2-hydroxyemodin 1-methylether. The only difference between the test compounds is the presence of a methoxy group at the C2 position. Aurantio-obtusin has a methoxy group at C2 position while 2-hydroxyemodin 1-methylether lacks it. Compared to their functional effect on V_1A_R, only aurantio-obtusin showed an antagonist effect with an IC_50_ value of 67.70 ± 2.41 µM. The antagonist effect of 2-hydroxyemodin 1-methylether on V_1A_R was 42.30 ± 9.89% at 100 µM concentration. A small change in the substituent at the C2 position showed a marked difference in the activity. Therefore, to further demonstrate the mechanism and clarify the reason behind the difference in activity, molecular docking simulation was conducted. As shown in Table 2, both the test compounds had a similar binding score and the same interacting residues at the active site of V_1A_R. However, the hydroxyl group at C6 and a methoxy group at C7 of aurantio-obtusin showed two additional H-bond interactions with the Ser341 residue. The same functional groups of aurantio-obtusin were responsible for H-bond interaction with key amino acid residues surrounding the catalytic cavity of human thrombin for inhibition [51]. Interaction with Ser341 was not observed in the case of 2-hydroxyemodin 1-methylether–V_1A_R binding. Consequently, it remains unclear whether Ser341 is responsible for the observed functional effect of aurantio-obtusin on V_1A_R.

Water extract from *Cassia obtusifolia* seeds reduced blood pressure in cold-induced hypertensive mice, modulated blood lipid contents, and improved pathological changes in renal structure [52]. Furthermore, the Cassia component, gluco-aurantio-obtusin exhibited good inhibition of angiotensin-converting enzyme (ACE) activity with an IC_50_ value of 30.24 ± 0.20 µM revealing its blood pressure regulating property while, its acid-hydrolyzed product aurantio-obtusin exhibited no activity [26].

A study on the effect of aurantio-obtusin on immunoglobulin E (IgE)-mediated allergic responses and LPS-induced RAW264.7 cells demonstrated suppression of degranulation, histamine production and ROS generation, inhibition of mRNA expression of TNF-α and IL-4, suppression of PGE2 production, and expression of COX-2 [25,53]. This demonstrates the benefits of aurantio-obtusin in treating allergy-related diseases. Likewise, aurantio-obtusin was reported for its larvicidal effect in *Anopheles gambiae* [54], inhibitory effect on IL-6 production in IL-1β-treated lung epithelial cells, A549, and attenuation of lung inflammatory responses in a mouse model of LPS-induced acute lung injury in male ICR mice [55], thereby revealing the therapeutic potential for treating inflammatory diseases [53]. Similarly, aurantio-obtusin stimulated chemotactic migration of MC3T3-E1 osteoblast cells and osteoblast differentiation and mineralization which are the therapeutic strategies to prevent osteoporosis and other metabolic bone diseases [29]. Additionally, aurantio-obtusin showed concentration-dependent vasorelaxation in phenylephrine precontracted rat mesenteric arteries rings via endothelial PI3K/Akt/eNOS pathway [56].

Since AVP levels in patients with heart failure and left ventricular (LV) dysfunction are often elevated [57,58,59], it is hypothesized that AVP might contribute to circulatory response in patients with heart failure and play a role in the development and progression of heart failure [60]. Moreover, the V_1A_R antagonist effect of aurantio-obtusin might be a promising approach for the treatment.

Transient brain ischemia/reperfusion injury occurs due to a temporary blockage of blood supply to the brain, and triggers selective neuronal loss/death in the most vulnerable brain region, especially the cornu ammonis 1 (CA1) field in the hippocampus [61,62,63]. Therefore, we further evaluated the neuroprotective effect of aurantio-obtusin in the transient brain ischemia/reperfusion injury C57BL/6 mice model. In the passive avoidance test, the BCCAO groups showed a significant reduction in latency time, however, treatment with aurantio-obtusin attenuated that reduction significantly (Figure 4). Likewise, compared to neuronal damage in the BCCAO groups, aurantio-obtusin significantly reduced the severity of damage in mCA1, dCA1, and cortex regions (Figure 6). However, treatment with 10 mg/kg aurantio-obtusin alone showed no toxicity which was comparable to the sham group. Overall, in vivo data depicts the neuroprotective effect of aurantio-obtusin. Additionally, the drug-likeness and ADME characteristics of aurantio-obtusin further support the possibility of drug development and optimization. However, whether the neuroprotective effect of aurantio-obtusin is regulated via V_1A_R remains unclear. This necessitates in-depth pharmacology of aurantio-obtusin using the V_1A_R deficit (V_1A_^−/−^) mice model along with detailed molecular dynamic studies.

This study evaluated the functional effect of major components from Cassia seeds—cassiaside and rubrofusarin gentiobioside along with the anthraquinone aurantio-obtusin and its substructure 2-hydroxyemodin 1-methylether. Based on the structure–activity relationship, additional interaction of the hydroxyl group at C6 and a methoxy group at C7 of aurantio-obtusin with the Ser341 residue was predicted functional for the observed V_1A_R antagonist effect. According to the previous study [64], aurantio-obtusin can cause hepatoxicity at a higher dose than 40 mg/kg in the rat. However, the dose applied for the rat needs verification through clinical trial, and demonstrating the optimal dose requires several clinical trial phases. In this study, a dose of 10 mg/kg was enough to exhibit a neuroprotective effect in C57BL/6 mice model. In addition, it was reported that aurantio-obtusin exist mainly in the form of metabolites such as sulfonation products and glucuronidation products in the body [65]. Therefore, further experiment should be conducted to find new active metabolites of auratio-obtusin, but also to unravel their pharmacokinetics and hepatotoxicity.

Altogether, this result highlights aurantio-obtusin as a V_1A_R antagonist and V_1A_R as a possible target for neuroprotection. However, in-depth in vivo studies on the V_1A_R deficit (V_1A_^−/−^) mice model is warranted to demonstrate the V_1A_R-regulated neuroprotection mechanism.

## 4. Materials and Methods

### 4.1. Chemicals and Reagents

Human endogenous (U373MG cells), murine interleukin-3 dependent pro-B (Ba/F3) and a transfected Chinese hamster ovary (CHO) cell lines were obtained from Eurofins Scientific (Le Bois I’Eveque, France). Buffers—Dulbecco’s modified Eagle medium (DMEM) buffer, 4-(2-hydroxyethyl)-1-piperazineethanesulfonic acid (HEPES) buffer and Hank’s balanced salt solution (HBSS) buffer—were purchased from Invitrogen (Carlsbad, CA, USA). The reference agonists dopamine, [Sar9, Met(O2)11]-SP, serotonin and arginine vasopressin, and antagonists (+) butaclamol, L 733060, (S)-WAY-100635, and [d(CH_2_)5^1^,Tyr(Me)_2_]-AVP) were obtained from Sigma-Aldrich (St. Louis, MO, USA). All other chemicals and reagents were purchased from Merck and Fluka, unless otherwise stated and were of highest available grade.

### 4.2. Plant Material

The raw seeds of *Cassia obtusifolia* Linn were purchased from Omni Herb Co. (Daegu, Korea), and authenticated by Prof. J.-H. Lee (Dongguk University, Gyeongju, Korea). A voucher specimen (no. 20130302) has been deposited in the laboratory of Prof. J. S. Choi.

### 4.3. Extraction, Fractionation and Isolation of Compounds

Extraction and fractionation of 3.0 kg dried seeds of *Cassia obtusifolia* was carried out as described previously [44] to obtain CH2Cl2 (107 g), EtOAc (147 g), and *n*-BuOH (76.8 g) fractions, respectively. The CH_2_Cl_2_ fraction (107 g) was subjected to silica gel column chromatography and eluted with CH_2_Cl_2_–MeOH (100:0→1:1, gradient to yield 10 subfractions (CF01 to CF10). CF08 (9.5 g) was further chromatographed on a silica gel column and eluted with *n*-hexane: EtOAc (10:1→10:1 gradient) to yield aurantio-obtusin (410 mg). Likewise, the EtOAc fraction (147 g) upon subjecting to silica gel column chromatography using CH_2_Cl_2_: MeOH (30:1→0:1 gradient) yielded 20 subfractions (EF01 to EF20). Repeated column chromatography of EF04 (2.2 g) using *n*-hexane: EtOAc (5:1→1:1 gradient) yielded 2-hydroxyemodin 1-methyl ether (68 mg). Subfraction EF07 (2.6 g) was chromatographed on a silica gel column and eluted with CH_2_Cl_2_–MeOH–H_2_O (15:1:0.1) to yield cassiaside (275 mg). Additionally, the 60% MeOH fraction (25.7 g) obtained by chromatographing 76.8 g of *n*-BuOH fraction and eluting with H_2_O-MeOH gradient solvent system was further chromatographed on a silica gel column and eluted with CH_2_Cl_2_–MeOH–H_2_O = 10:1:0.1 to yield 11 subfractions (B60M01–B60M11). Fraction B60M03 gave precipitate (9.0 g) which was dissolved in MeOH–H_2_O (2:1), chromatographed on a silica gel column and eluted with EtOAc–MeOH–H_2_O (24:3:2) to rubrofusarin gentiobioside (76 mg).

All the isolated compounds were identified by comparing the spectral data from the literature [26,39,44,66] and the purity of each compounds was estimated to be >98% based on spectral data.

Cassiaside: Yellowish powder, ^1^H-NMR (600 MHz, DMSO-*d*_6_) δ: 10.32 (OH), 7.06 (1H, s, H-10), 6.72 (1H, d, *J* = 1.6 Hz, H-9), 6.68 (1H, d, *J* = 1.9 Hz, H-7), 6.15 (1H, s, H-3), 4.97 (1H, d, *J* = 7.5 Hz, H-1′), 2.37 (3H, s, CH_3_); ^13^C-NMR (150 MHz, DMSO-*d*_6_) δ: 180.67 (C-4), 168.58 (C-2), 162.07 (C-5), 159.69 (C-8), 158.26 (C-6), 152.24 (C-11), 140.42 (C-14), 106.86 (C-3), 106.50 (C-13), 103.00 (C-12), 102.46 (C-9), 101.59 (C-7), 101.26 (C-1′), 99.91 (C-10), 77.22 (C-5′), 76.37 (C-3′), 73.49 (C-2′), 69.53 (C-4′), 60.61 (C-6′), and 20.11 (CH_3_).

Aurantio-obtusin: Orange needles, ^1^H-NMR (600 MHz, DMSO-*d*_6_) δ: 10.58 (OH), 7.74 (1H, s, H-4), 7.14 (1H, d, *J* = 1.9 Hz, H-5), 3.82 (3H, s, OCH_3_), 3.79 (3H, s, OCH_3_), 2.26 (3H, s, CH_3_). ^13^C-NMR (150 MHz, DMSO-*d*_6_) δ: 187.10 (C-9), 180.34 (C-10), 156.92 (C-8), 156.70 (C-6), 155.50 (C-2), 147.19 (C-1), 139.38 (C-7), 131.94 (C-3), 128.48 (C-11), 125.83 (C-4), 124.82 (C-14), 123.68 (C-13), 111.01 (C-12), 107.70 (C-5), 61.17 (OCH_3_), 59.96 (OCH_3_), and 16.44 (CH_3_).

2-Hydroxyemodin 1-methylether: Orange needles, ^1^H-NMR (400 MHz, DMSO-*d*_6_) δ: 10.71 (OH), 7.75 (1H, s, H-4), 7.02 (1H, d, *J* = 2.2 Hz, H-5), 6.52 (1H, d, *J* = 2.4 Hz, H-7), 3.78 (3H, s, OCH_3_), 2.26 (3H, s, CH_3_); ^13^C-NMR (100 MHz, DMSO-*d*_6_) δ: 186.52 (C-9), 180.85 (C-10), 164.60 (C-8), 164.56 (C-6), 155.79 (C-2), 147.18 (C-1), 134.53 (C-11), 131.85 (C-3), 125.95 (C-4), 124.80 (C-14), 123.65 (C-13), 110.17 (C-12), 107.50 (C-7), 107. 21 (C-5), 61.18 (OCH_3_), and 16.45 (CH_3_).

Rubrofusarin 6-O-β-gentiobioside: Yellow needles, ^1^H-NMR (600 MHz, DMSO-*d*_6_) δ: 7.17 (lH, s, H-10), 6.93 (1H, d, *J* = 2.0 Hz, H-9), 6.79 (1H, d, *J* = 2.7 Hz, H-7), 6.17 (1H, s, H-3), 5.06 (lH, d, *J* = 7.6 Hz, glycosyl H-l), 4.20 (1H, d, *J* = 7.6 Hz, glycosyl H-l), 3.87 (3H, s, OCH_3_) and 2.38 (3H, s, CH_3_). ^13^C-NMR (150 MHZ, DMSO-*d*_6_) δ: 183.80 (C-4), 168.90 (C-2), 161.90 (C-5), 161.10 (C-8), 157.60 (C-6), 152.40 (C-11), 140.30 (C-14), 107.70 (C-13), 106.7 (C-3), 103.60 (C-10), 103.60 (C-1′), 101.10(C-12), 100.90 (C-7), 100.70 (C-1′′), 99.70 (C-9), 76.90 (C-3′), 76.60 (C-5′′), 76.40 (C-3′′), 75.50 (C-5′), 73.57 (C-2′), 73.50 (C-2′′), 70.12 (C-4′), 69.64 (C-4′′), 68.70 (C-6′), 61.09 (C-6′′), 55.50 (OCH_3_), and 20.20 (CH_3_).

### 4.4. GPCR Functional Assay

Cell based functional GPCR assays were conducted at Eurofins Cerep (Le Bois I’Eveque, France) using transfected cells expressing human cloned receptors namely dopamine (D_3_R), serotonin (5-HT_1A_R), tachykinin (NK_1_R), and vasopressin (V_1A_R). Agonist/antagonist effect of test compounds in each receptor was evaluated by measuring the level of secondary messengers.

### 4.5. Measurement of cAMP Level

The effect of test compounds on hD_3_ receptor expressed in CHO cells was evaluated by measuring their effect on cAMP modulation using HTRF detection. In brief, a plasmid containing the GPCR gene of interest (dopamine D3) was transfected into Chinese hamster ovary (CHO) cells. The resulting stable transfectants (CHO-GPCR cells line) were suspended in HBSS buffer (Invitrogen, Carlsbad, CA, USA) supplemented with 20 mM HEPES buffer and 500 μM IBMX. The solutions were distributed into microplates at a density of 5 × 10^3^ cells/well and incubated for 30 min at room temperature (RT) in the absence (control) or presence of aurantio-obtusin (100 µM) or reference agonist. Cells were lysed and a fluorescence acceptor (D3-labeled cAMP) and fluorescence donor (anti-cAMP antibody with europium cryptate) were added following the incubation. The fluorescence transfer was measured (λex = 337 nm and λem = 620 or 665 nm) using a microplate reader (Envision, PerkinElmer, Waltham, MA, USA) after 60 min at RT. Results are expressed as a percentage of the control response to dopamine for the agonist effect and as percent inhibition of the control response to dopamine. The standard reference control was dopamine [67]. Cellular agonist effect was calculated as the percentage of the control response to 300 nM dopamine for D3R, and cellular antagonist effect was calculated as the percentage inhibition of agonist response of 10 nM dopamine. To validate the result, reference antagonist (+)-butaclamol was used for D_3_R.

### 4.6. Measurement of Intracellular Ca^2+^ Ion Concentration

Functional effect of test compounds on the h5-HT_1A_R, hNK_1_R, and hV_1A_R was evaluated fluorimetrically by measuring intracellular Ca^2+^ concentration. Agonist activity of test compounds on the h5-HT_1A_R expressed in Ba/F3 cells, hNK_1_R expressed in U373MG cells, and V_1A_R expressed in transfected CHO cells was determined by measuring their effect on cytosolic Ca^2+^ ion mobilization using a fluorimetric detection method described in our previous reports [68,69]. For antagonist activity, the effect on agonist-induced cytosolic Ca^2+^ ion mobilization was measured. Cellular agonist effect at h5-HT_1A_R was calculated as the percentage of the control response to serotonin (2.5 μM), and antagonist effect was calculated as the percentage inhibition of the control response to 30 nM serotonin. To validate the result, reference antagonist (S)-WAY-100635 was employed. Likewise, for the cellular agonist effect at hNK_1_R, the percentage of the control response to 30 nM [Sar9, Met(O2)11]-SP was determined and for antagonist effect, percentage inhibition of control response to 1 nM [Sar9, Met(O2)11]-SP was recorded. The standard reference antagonist L 733,060 was used to validate the result. Additionally, for the cellular agonist at hV_1A_R, the percentage of the control response to 1 μM AVP was determined and for antagonist effect, percentage inhibition of control response to 10 nM AVP was recorded. The standard reference antagonist [d(CH_2_)5^1^,Tyr(Me)_2_]-AVP was used to validate the result.

### 4.7. Homology Modeling of V_1A_R

The primary sequence of the human V_1A_R was obtained from UniProt (ID: P37288). µ-Opioid receptor obtained from RCSB protein data bank (PDB) with ID of 4DKL was used as a template for homology modeling of V_1A_R. Modeling was conducted using SWISS-MODEL and refined in ModRefiner server (RMSD = 0.645 Å) [70].

### 4.8. Molecular Docking

To get insight of reciprocal interactions between compounds and the target, docking simulation was conducted using AutoDock 4.2. program [71]. 3D structure of aurantio-obtusin and 2-hydroxyemodin 1-methylether were constructed using Chem3D Pro v12.0 and refined using Discovery Studio (v17.2, Accelrys, San Diego, CA, USA). To assess the appropriate binding conformation of the ligands with protein target, AutoDockTools (ADT) was used to conduct docking simulation. For the docking calculations, Gasteiger charges were added by default, the rotatable bonds were set by ADT and all torsions were allowed to rotate. The grid maps were generated using AutoGrid. The docking protocol for rigid and flexible ligand docking included 10 independent genetic algorithms. The results were analyzed and visualized using Discovery Studio.

### 4.9. Drug-Likeness and ADME Prediction

Drug-likeness prediction was carried out with PreADMET (v2.0, YONSEI University, Seoul, Korea). This web-based server can be used to predict absorption, distribution, metabolism, and excretion (ADME) data and build a drug-likeness library in silico.

### 4.10. Animal

Male C57BL/6 mice (22–26 g, 7 weeks) were purchased from the Orient Co. Ltd., a branch of Charles River Laboratories (Seoul, Korea), and kept in the University Animal Care Unit for 1 week prior to the experiments. The animals were housed five per cage, allowed access to water and food ad libitum; the environment was maintained at a constant temperature (23 ± 1 °C) and humidity (60 ± 10%) under a 12-h light/dark cycle (the lights were on from 07:30 to 19:30 h). Forty mice were divided equally into four groups (sham + vehicle, *n* = 10; sham + drug, *n* = 10; bilateral common carotid artery occluded ischemia + vehicle, *n* = 10; bilateral common carotid artery occluded ischemia + drug, *n* = 10) for experiment. The treatment and maintenance of the animals were carried out in accordance with the Animal Care and Use Guidelines of Dong-A University, Korea. All in vivo experiments were performed according to the protocols approved by the Institutional Animal Care and Use Committee of Dong-A University (approved protocol numbers: DIACUC-approved-17-20) and were in accordance with the National Institutes of Health guidelines.

### 4.11. Transient Forebrain Ischemia Surgery

C57BL/6 mice were anesthetized with 2.0% isoflurane and 70% nitrous oxide in oxygen and subjected to transient forebrain ischemia. The transient forebrain ischemia was induced by bilateral common carotid artery occlusion (BCCAO) with aneurysm clips for 20 min, and the circulation was restored by removing the clips. Mice that received the same surgical operation without carotid artery clipping served as sham-operated controls. During the surgical procedure, the rectal temperature was maintained at 37 ± 0.5 °C with a heating pad (Biomed S.L., Barcelona, Spain). The regional cerebral blood flow (rCBF) was monitored using laser Doppler flowmetry (Perimed, PF5010, JarFalla, Sweden). The mice that showed between 80% and 95% reduction of rCBF were used in the study. After reperfusion, the animals were placed in a warm incubator (32–33 °C) and returned to their home cages. Aurantio-obtusin, which was dissolved in 10% Tween 80 solution, was administered from 1 h to 7 days after BCCAO (10 mg/kg, p.o., once daily).

### 4.12. Passive Avoidance Test

Passive avoidance test was conducted 1 h after the last drug administration. The animals underwent two separated trials, an initial training trial and a test trial 24 h later. For the training trial, a mouse was initially placed in the light compartment, and the door between the two compartments was opened 10 s later. When the mouse entered the dark compartment, the guillotine door automatically closed and an electrical foot shock (0.5 mA, 3 s) was delivered through the floor. For the retention trial, the mouse was again placed in the light compartment and the time required to enter the dark compartment was recorded.

### 4.13. Slices Preparation and Nissl Staining

One day after the test trial of passive avoidance test, mice were anesthetized with Zoletil 50^®^ (10 mg/kg, i.m.) and then perfused transcardially with a 100 mM phosphate buffer (pH 7.4) followed by ice-cold 4% paraformaldehyde. The brains of the mice were removed and post-fixed in a phosphate buffer (50 mM, pH 7.4) containing 4% paraformaldehyde overnight, then immersed in a 30% sucrose solution (in 50 mM phosphate-buffered saline, PBS), and stored at 4 °C until sectioned. The frozen brains were coronally sectioned on a cryostat at 30 μm and then stored in a storage solution (30% ethylene glycol, 30% glycerin, and 20 mM phosphate buffer) at 4 °C. Hippocampal sections were collected based on the mouse brain atlas.

After the sections were mounted onto gelatin-coated slides, they were stained with 0.5% cresyl violet, dehydrated through graded alcohols (70, 80, 90, and 100% × 2), placed in xylene, and covered with a coverslip after the addition of Histomount media. The number of cells in selected regions (medial CA1, mCA1; dorsal CA1, dCA1; CA2; cortex) were determined using a computerized image analysis system (Leica Microsystems AG, Wetzlar, Germany). The cells were counted in six sections by every eight sections interval (total 48 sections) per animal by a person blind to the treatment group, and the average cell count per section was computed. The degree of damage by the Nissl staining after ischemia was semiquantitatively scored from 0 to 3 (0, normal; 1, <30% of the neurons were irreversibly damaged; 2, 30–60% of the neurons were irreversibly damaged; 3, 60–100% of the neurons were irreversibly damaged).

### 4.14. Statistical Analysis

Statistical analysis was performed by Student’s *t*-test using Microsoft Excel 2016 (Microsoft Corporation, Redmond, WA, USA). All experiments were carried out in triplicate on three individual days and are expressed as the mean ± standard deviation (SD). Results of Nissl staining and passive avoidance test were analyzed using one-way ANOVA (GraphPad Prism ver. 9). Data are expressed as the mean ± SD with raw data. * *p* < 0.05.

## Figures and Tables

**Table 1:** Efficacy values (% stimulation and % inhibition) of Cassia compounds on various GPCRs at 100 µM.

Receptors	Rubrofusarin Gentiobioside	Cassiaside	Aurantio-Obtusin	Reference Drugs for Target Receptors	
	% Stimulation ^a^ (% Inhibition ^b^)	% Stimulation ^a^ (% Inhibition ^b^)	% Stimulation ^a^ (% Inhibition ^b^)	Agonist Effect: EC_50_^c^; Antagonist Effect: (IC_50_^d^)	
D_3_ (h)	7.05 ± 1.63 (−0.05 ± 0.78)	14.75 ± 1.20 (−5.05 ± 2.05)	33.00 ± 1.84 (−2.30 ± 0.57)	Dopamine	2.7
				(+) butaclamol	(25)
NK_1_ (h)	−1.40 ± 0.42 (3.90 ± 5.94)	−1.70 ± 0.14 (20.50 ± 1.84)	−6.40 ± 0.00 (47.60 ± 3.11)	[Sar9, Met(O2)11]-SP	0.18
				L 733,060	(0.58)
5-HT_1A_ (h)	−0.30 ± 0.28 (4.25 ± 2.47)	1.75 ± 0.07 (−13.45 ± 0.07)	−6.10 ± 0.00 (10.10 ± 4.10)	Serotonin	1.6
				(S)-WAY-100635	(5.6)
V_1A_ (h)	−4.40 ± 1.41 (−13.60 ± 0.57)	1.40 ± 2.83 (−9.90 ± 6.65)	−33.20 ± 2.69 (71.80 ± 6.08)	Arginine vasopressin	0.89
				d(CH_2_)5^1^,Tyr(Me)_2_]-AVP	(4.5)

^a^ % Stimulation (agonist effect) and ^b^ % inhibition (antagonist effect) of control agonist response at 100 µM of test compounds. ^c^ EC_50_ (nM) values of standard agonist. ^d^ IC_50_ (nM) values of standard antagonists. Compounds were screened at 100 µM concentration to evaluate the functional effect on various receptors. Values in brackets for test samples represent the percentage inhibition of control agonist response while that of reference drugs represent the 50% inhibition concentration. Values are expressed as mean ± SD of triplicate experiment.

**Table 2:** The binding affinity of aurantio-obtusin, 2-hydroxyemodin 1-methylether, and reference ligands with human V_1A_ receptor using AutoDock 4.2.

Ligand	Binding Score (kcal/mol)	Interacting Residues		
		H-Bond	Electrostatic	Hydrophobic
Aurantio-obtusin	−7.58	Ala101, Lys128, Ser341, Gln131	−	Val100(π-σ), Ala101(π-alkyl), Trp304(π-π T-shaped, π-alkyl), Ala334(alkyl, π-alkyl), Met135(alkyl, π-aAlkyl), Val100(alkyl), Phe307(π-alkyl)
2-Hydroxyemodin 1-methylether	−7.34	Ala101, Lys128, Gln131, Gly337	−	Val100(π-σ), Trp304(π-π T-shaped), Phe307(π-π T-shaped, π-alkyl), Ala334(alkyl, π-alkyl), Met135(alkyl, π-alkyl), Ala101(π-alkyl)
d(CH_2_)5^1^,Tyr(Me)_2_]-AVP ^a^ (antagonist)	−6.07	Lys128, Asn327, Asp112, Thr331, Gln131	−	Trp332(π-π stacked), Ile330(alkyl), Tyr115(π-alkyl)
AVP ^a^ (agonist)	−8.62	Asp202, Glu54, Asp112, Thr331, Thr198	Trp204(π-Sulfur), Trp111(π-Sulfur)	Ile330(Alkyl), Val194(π-Alkyl)

^a^ Arginine vasopressin (AVP) and d(CH_2_)5^1^,Tyr(Me)_2_]-AVP were used as reference agonist and antagonist, respectively.

**Table 3:** Drug-likeness and absorption, distribution, metabolism, and excretion (ADME) characteristics as predicted by PreADMET.

Compounds	Drug-Likeness ^g^		ADME Characteristics					
	MDDR-like rule	Lipinski’s rule	Log Po/w ^a^	PPB ^b^	HIA ^c^	In vitro MDCK cell permeability (nm/s) ^d^	In vitro Caco2 permeability (nm/s) ^e^	In vivo BBB penetration ([brain]/[blood]) ^f^
Aurantio-obtusin	Mid-structure	Suitable	2.53	86.98	84.66	113.20	19.17	0.48

^a^ The log of the coefficient of solvent partitioning between 1-octanol and water (the lipophilicity values log*P*/log*D* ranging from 1.7 to 2.8 demonstrate the highest CNS penetration). ^b^ Plasma protein binding (PPB) (<90% represents weak binding and >90% represents strong binding). ^c^ Human intestinal absorption (HIA) (0–20% is poorly absorbed, 20–70% is moderately absorbed and 70–100% is well absorbed). ^d^ Permeability across Madin–Darby Canine Kidney (MDCK) cells. ^e^ Permeability across human epithelial colorectal adenocarcinoma (Caco2) cells (0–10 nm/s is low permeability, 10–100 nm/s is medium permeability, and >100 nm/s is high permeability). ^f^ Absorption by the CNS (value < 0.1 is low absorption by the central nervous system, 0.1–2.0 is middle absorption, and >2.0 is high absorption). ^g^ Lipinski’s rule: an orally active drug has no more than one violation of H-bond donors (≤5), H-bond acceptors (≤10), molecular weight (≤500 Da), and log *P* (≤5). MDDR-like rule: the MDDR-like rule describes a molecule as drug-like or non-drug-like based on the number of rings, rigid bonds, and rotatable bonds.

## Data Availability

The data presented in this study are available within the article.

## References

[1] Jobst A., Dehning S., Ruf S., Notz T., Buchheim A., Henning-Fast K., Meißner D., Meyer S., Bondy B., Müller N. (2014). Oxytocin and vasopressin levels are decreased in the plasma of male schizophrenia patients. Acta Neuropsychiatr..

[2] Yang S.Y., Cho S.-C., Yoo H.J., Cho I.H., Park M., Kim B.-N., Kim J.-W., Shin M.-S., Park T.-W., Son J.-W. (2010). Association study between single nucleotide polymorphisms in promoter region of AVPR1A and Korean autism spectrum disorders. Neurosci. Lett..

[3] LoParo D., Waldman I. (2015). The oxytocin receptor gene (OXTR) is associated with autism spectrum disorder: A meta-analysis. Mol. Psychiatry.

[4] Schnider P., Bissantz C., Bruns A., Dolente C., Goetschi E., Jakob-Roetne R., Künnecke B., Mueggler T., Muster W., Parrott N. (2020). Discovery of balovaptan, a vasopressin 1a receptor antagonist for the treatment of autism spectrum disorder. J. Med. Chem..

[5] Yuen K.W., Garner J.P., Carson D.S., Keller J., Lembke A., Hyde S.A., Kenna H.A., Tennakoon L., Schatzberg A.F., Parker K.J. (2014). Plasma oxytocin concentrations are lower in depressed vs. healthy control women and are independent of cortisol. J. Psychiatr. Res..

[6] Simon N.G., Guillon C., Fabio K., Heindel N.D., Lu S.-F., Miller M., Ferris C.F., Brownstein M.J., Garripa C., Koppel G.A. (2008). Vasopressin antagonists as anxiolytics and antidepressants: Recent developments. Recent Pat. CNS Drug Discov..

[7] Albers H.E. (2012). The regulation of social recognition, social communication and aggression: Vasopressin in the social behavior neural network. Horm. Behav..

[8] Benarroch E.E. (2013). Oxytocin and vasopressin: Social neuropeptides with complex neuromodulatory functions. Neurology.

[9] Bielsky I.F., Hu S.-B., Szegda K.L., Westphal H., Young L.J. (2003). Profound impairment in social recognition and reduction in anxiety-like behavior in vasopressin V1a receptor knockout mice. Neuropsychopharmacology.

[10] Ferris C.F., Lu S.-F., Messenger T., Guillon C.D., Heindel N., Miller M., Koppel G., Robert Bruns F., Simon N.G. (2006). Orally active vasopressin V1a receptor antagonist, SRX251, selectively blocks aggressive behavior. Pharmacol. Biochem. Behav..

[11] Jorgensen W.T., Gulliver D.W., Katte T.A., Werry E.L., Reekie T.A., Connor M., Kassiou M. (2018). Conformationally rigid derivatives of WAY-267,464: Synthesis and pharmacology at the human oxytocin and vasopressin-1a receptors. Eur. J. Med. Chem..

[12] Vakili A., Kataoka H., Plesnila N. (2005). Role of arginine vasopressin V1 and V2 receptors for brain damage after transient focal cerebral ischemia. J. Cereb. Blood Flow Metab..

[13] Dou D., Chen L., Di H., Song Z., Li S., Bu X., Dai Q., Wang S., Li J.X., Zhu X. (2019). Vasopressin augments TNBS-induced colitis through enteric neuronal V1a receptor-mediated COX-2-dependent prostaglandin release from mast cells in mice. Neurogastroenterol. Motil..

[14] Linas S.L., Anderson R.J., Guggenheim S.J., Robertson G.L., Berl T., Dickmann D.C. (1981). Role of vasopressin in impaired water excretion in conscious rats with experimental cirrhosis. Kidney Int..

[15] Liu X., Luo G., Jiang J., Ma T., Lin X., Jiang L., Cheng J., Tao R. (2016). Signaling through hepatocyte vasopressin receptor 1 protects mouse liver from ischemia-reperfusion injury. Oncotarget.

[16] Narayen G., Mandal S.N. (2012). Vasopressin receptor antagonists and their role in clinical medicine. Indian J. Endocrinol. Metab..

[17] Share L. (1988). Role of vasopressin in cardiovascular regulation. Physiol. Rev..

[18] Aoyagi T., Koshimizu T.-A., Tanoue A. (2009). Vasopressin regulation of blood pressure and volume: Findings from V1a receptor–deficient mice. Kidney Int..

[19] Morel A., O’Carroll A.-M., Brownstein M.J., Lolaft S.J. (1992). Molecular cloning and expression of a rat V1a arginine vasopressin receptor. Nature.

[20] Palin K., Moreau M.L., Sauvant J., Orcel H., Nadjar A., Duvoid-Guillou A., Dudit J., Rabié A., Moos F. (2009). Interleukin-6 activates arginine vasopressin neurons in the supraoptic nucleus during immune challenge in rats. Am. J. Physiol. Endocrinol. Metab..

[21] Raber J., Bloom F.E. (1994). IL-2 induces vasopressin release from the hypothalamus and the amygdala: Role of nitric oxide-mediated signaling. J. Neurosci..

[22] Bolognani F., Del Valle Rubido M., Squassante L., Wandel C., Derks M., Murtagh L., Sevigny J., Khwaja O., Umbricht D., Fontoura P. (2019). A phase 2 clinical trial of a vasopressin V1a receptor antagonist shows improved adaptive behaviors in men with autism spectrum disorder. Sci. Transl. Med..

[23] Koshimizu T.-A., Nasa Y., Tanoue A., Oikawa R., Kawahara Y., Kiyono Y., Adachi T., Tanaka T., Kuwaki T., Mori T. (2006). V1a vasopressin receptors maintain normal blood pressure by regulating circulating blood volume and baroreflex sensitivity. Proc. Natl. Acad. Sci. USA.

[24] Huijuan S., Zhuju W., Liying T. (2011). Simultaneous determination of 4 major components in semen cassiae obtusifoline by HPLC. Zhongguo Zhong Yao Za Zhi.

[25] Kim M., Lim S.J., Lee H.-J., Nho C.W. (2015). Cassia tora seed extract and its active compound aurantio-obtusin inhibit allergic responses in IgE-mediated mast cells and anaphylactic models. J. Agric. Food Chem..

[26] Hyun S.K., Lee H., Kang S.S., Chung H.Y., Choi J.S. (2009). Inhibitory activities of *Cassia tora* and its anthraquinone constituents on angiotensin-converting enzyme. Phytother. Res..

[27] Byun E., Jeong G.-S., An R.-B., Li B., Lee D.-S., Ko E.-K., Yoon K.-H., Kim Y.-C. (2007). Hepatoprotective compounds of Cassiae Semen on tacrine-induced cytotoxicity in Hep G2 cells. Korean J. Pharmacogn..

[28] Choi J.S., Lee H.J., Park K.-Y., Ha J.-O., Kang S.S. (1997). In vitro antimutagenic effects of anthraquinone aglycones and naphthopyrone glycosides from *Cassia tora*. Planta Med..

[29] Vishnuprasad C.N., Tsuchiya T., Kanegasaki S., Kim J.H., Han S.S. (2014). Aurantio-obtusin stimulates chemotactic migration and differentiation of MC3T3-E1 osteoblast cells. Planta Med..

[30] El-Halawany A.M., Chung M.H., Nakamura N., Ma C.-M., Nishihara T., Hattori M. (2007). Estrogenic and anti-estrogenic activities of *Cassia tora* phenolic constituents. Chem. Pharm. Bull..

[31] Jung H.A., Ali M.Y., Jung H.J., Jeong H.O., Chung H.Y., Choi J.S. (2016). Inhibitory activities of major anthraquinones and other constituents from *Cassia obtusifolia* against β-secretase and cholinesterases. J. Ethnopharmacol..

[32] Kim D.H., Yoon B.H., Kim Y.-W., Lee S., Shin B.Y., Jung J.W., Kim H.J., Lee Y.S., Choi J.S., Kim S.Y. (2007). The seed extract of *Cassia obtusifolia* ameliorates learning and memory impairments induced by scopolamine or transient cerebral hypoperfusion in mice. J. Pharmacol. Sci..

[33] Shrestha S., Seong S.H., Paudel P., Jung H.A., Choi J.S. (2017). Structure related inhibition of enzyme systems in cholinesterases and BACE1 in vitro by naturally occurring naphthopyrone and its glycosides isolated from *Cassia obtusifolia*. Molecules.

[34] Yi J.H., Park H.J., Lee S., Jung J.W., Kim B.C., Lee Y.C., Ryu J.H., Kim D.H. (2016). *Cassia obtusifolia* seed ameliorates amyloid β-induced synaptic dysfunction through anti-inflammatory and Akt/GSK-3β pathways. J. Ethnopharmacol..

[35] Ravi S.K., Narasingappa R.B., Joshi C.G., Girish T.K., Vincent B. (2018). Neuroprotective effects of *Cassia tora* against paraquat-induced neurodegeneration: Relevance for Parkinson’s disease. Nat. Prod. Res..

[36] Jung H.A., Ali M.Y., Choi J.S. (2017). Promising inhibitory effects of anthraquinones, naphthopyrone, and naphthalene glycosides, from *Cassia obtusifolia* on α-glucosidase and human protein tyrosine phosphatases 1B. Molecules.

[37] Shrestha S., Paudel P., Seong S.H., Min B.S., Seo E.K., Jung H.A., Choi J.S. (2018). Two new naphthalenic lactone glycosides from *Cassia obtusifolia* L. seeds. Arch. Pharm. Res..

[38] Ali M.Y., Jannat S., Jung H.A., Min B.S., Paudel P., Choi J.S. (2018). Hepatoprotective effect of *Cassia obtusifolia* seed extract and constituents against oxidative damage induced by *tert*-butyl hydroperoxide in human hepatic HepG2 cells. J. Food Biochem..

[39] Paudel P., Jung H.A., Choi J.S. (2018). Anthraquinone and naphthopyrone glycosides from *Cassia obtusifolia* seeds mediate hepatoprotection via Nrf2-mediated HO-1 activation and MAPK modulation. Arch. Pharm. Res..

[40] Yen G.-C., Chen H.-W., Duh P.-D. (1998). Extraction and identification of an antioxidative component from Jue Ming Zi (*Cassia tora* L.). J. Agri. Food Chem..

[41] Choi J.S., Lee H.J., Kang S.S. (1994). Alatemin, cassiaside and rubrofusarin gentiobioside, radical scavenging principles from the seeds of *Cassia tora* on 1, 1-diphenyl-2-picrylhydrazyl (DPPH) radical. Arch. Pharm. Res..

[42] Dong X., Fu J., Yin X., Yang C., Zhang X., Wang W., Du X., Wang Q., Ni J. (2017). Cassiae semen: A review of its phytochemistry and pharmacology. Mol. Med. Rep..

[43] Drever B.D., Anderson W.G., Riedel G., Kim D.H., Ryu J.H., Choi D.-Y., Platt B. (2008). The seed extract of *Cassia obtusifolia* offers neuroprotection to mouse hippocampal cultures. J. Pharmacol. Sci..

[44] Paudel P., Seong S.H., Shrestha S., Jung H.A., Choi J.S. (2019). In vitro and in silico human monoamine oxidase inhibitory potential of anthraquinones, naphthopyrones, and naphthalenic lactones from *Cassia obtusifolia* Linn seeds. ACS Omega.

[45] Paudel P., Seong S.H., Fauzi F.M., Bender A., Jung H.A., Choi J.S. (2020). Establishing GPCR targets of hMAO active anthraquinones from *Cassia obtusifolia* Linn seeds using in silico and in vitro methods. ACS Omega.

[46] Oprea T.I. (2000). Property distribution of drug-related chemical databases. J. Comput. Aided Mol. Des..

[47] Lipinski C.A., Lombardo F., Dominy B.W., Feeney P.J. (1997). Experimental and computational approaches to estimate solubility and permeability in drug discovery and development settings. Adv. Drug Deliv. Rev..

[48] Misra A., Ganesh S., Shahiwala A., Shah S.P. (2003). Drug delivery to the central nervous system: A review. J. Pharm. Pharm. Sci..

[49] Baell J.B. (2016). Feeling Nature’s PAINS: Natural Products, Natural Product Drugs, and Pan Assay Interference Compounds (PAINS). J. Nat. Prod..

[50] Baell J.B., Holloway G.A. (2010). New substructure filters for removal of pan assay interference compounds (PAINS) from screening libraries and for their exclusion in bioassays. J. Med. Chem..

[51] Yu X., Wei L.-H., Zhang J.-K., Chen T.-R., Jin Q., Wang Y.-N., Zhang S.-J., Dou T.-Y., Cao Y.-F., Guo W.-Z. (2019). Anthraquinones from Cassiae semen as thrombin inhibitors: In vitro and in silico studies. Phytochemistry.

[52] Pan Z.-J., Lu Q., Pan L., Xu X.-X., Pang J.-S. (2010). Effects of water extracts from *Cassia obtusifolia* on blood pressure, blood lipid and renal structure in cold-induced hypertensive mice. Chin. J. Exp. Tradit. Med Formulae.

[53] Hou J., Gu Y., Zhao S., Huo M., Wang S., Zhang Y., Qiao Y., Li X. (2018). Anti-inflammatory effects of aurantio-obtusin from seed of *Cassia obtusifolia* L. through modulation of the NF-κB pathway. Molecules.

[54] Mbatchou V.C., Tchouassi D.P., Dickson R.A., Annan K., Mensah A.Y., Amponsah I.K., Jacob J.W., Cheseto X., Habtemariam S., Torto B. (2017). Mosquito larvicidal activity of *Cassia tora* seed extract and its key anthraquinones aurantio-obtusin and obtusin. Parasit. Vectors.

[55] Kwon K.S., Lee J.H., So K.S., Park B.K., Lim H., Choi J.S., Kim H.P. (2018). Aurantio-obtusin, an anthraquinone from cassiae semen, ameliorates lung inflammatory responses. Phytother. Res..

[56] Li S., Li Q., Lv X., Liao L., Yang W., Li S., Lu P., Zhu D. (2015). Aurantio-obtusin relaxes systemic arteries through endothelial PI3K/AKT/eNOS-dependent signaling pathway in rats. J. Pharmacol. Sci..

[57] Riegger G.A., Liebau G., Kochsiek K. (1982). Antidiuretic hormone in congestive heart failure. Am. J. Med..

[58] Goldsmith S.R., Francis G.S., Cowley A.W., Levine T.B., Cohn J.N. (1983). Increased plasma arginine vasopressin levels in patients with congestive heart failure. J. Am. Coll. Cardiol..

[59] Creager M.A., Faxon D.P., Cutler S.S., Kohlmann O., Ryan T.J., Gavras H. (1986). Contribution of vasopressin to vasoconstriction in patients with congestive heart failure: Comparison with the renin-angiotensin system and the sympathetic nervous system. J. Am. Coll. Cardiol..

[60] Udelson J.E., Smith W.B., Hendrix G.H., Painchaud C.A., Ghazzi M., Thomas I., Ghali J.K., Selaru P., Chanoine F., Pressler M.L. (2001). Acute hemodynamic effects of conivaptan, a dual V1A and V2 vasopressin receptor antagonist, in patients with advanced heart failure. Circulation.

[61] Pulsinelli W.A. (1985). Selective neuronal vulnerability: Morphological and molecular characteristics. Prog. Brain Res..

[62] Kirino T. (1982). Delayed neuronal death in the gerbil hippocampus following ischemia. Brain Res..

[63] Pulsinelli W.A., Brierley J.B., Plum F. (1982). Temporal profile of neuronal damage in a model of transient forebrain ischemia. Ann. Neurol..

[64] Xu L., Li J., Tang X., Wang Y., Ma Z., Gao Y. (2019). Metabolomics of aurantio-obtusin-induced hepatotoxicity in rats for discovery of potential biomarkers. Molecules.

[65] Xiao S.-L., Guan L.-J., Jiang R.-F., Wang X.-G., Li X., Cai W. (2020). The metabolism and pharmacokinetics of rhein and aurantio-obtusin. Curr. Drug Metab..

[66] Choi B.S., Kim Y.J., Choi J.S., Lee H.J., Lee C.J. (2019). Obtusifolin isolated from the seeds of *Cassia obtusifolia* regulates the gene expression and production of MUC5AC mucin in airway epithelial cells via affecting NF-κB pathway. Phytother. Res..

[67] Paudel P., Seong S.H., Jung H.A., Choi J.S. (2019). Characterizing fucoxanthin as a selective dopamine D3/D4 receptor agonist: Relevance to Parkinson’s disease. Chem. Biol. Interact..

[68] Paudel P., Seong S.H., Wu S., Park S., Jung H.A., Choi J.S. (2019). Eckol as a potential therapeutic against neurodegenerative diseases targeting dopamine D3/D4 receptors. Mar. Drugs.

[69] Seong S.H., Paudel P., Choi J.-W., Ahn D.H., Nam T.-J., Jung H.A., Choi J.S. (2019). Probing multi-target action of phlorotannins as new monoamine oxidase inhibitors and dopaminergic receptor modulators with the potential for treatment of neuronal disorders. Mar. Drugs.

[70] Xu D., Zhang Y. (2011). Improving the physical realism and structural accuracy of protein models by a two-step atomic-level energy minimization. Biophys. J..

[71] Goodsell D.S., Morris G.M., Olson A.J. (1996). Automated docking of flexible ligands: Applications of AutoDock. J. Mol. Recognit..

